# Dynamic Regulatory Processes in the Transition From Suicidal Ideation to Action in Adults Leaving Inpatient Psychiatric Care: Protocol for an Intensive Longitudinal Study

**DOI:** 10.2196/38582

**Published:** 2022-06-30

**Authors:** Sarah E Victor, Kirsten Christensen, Sheri L Johnson, Jason Van Allen, Leslie A Brick

**Affiliations:** 1 Department of Psychological Sciences Texas Tech University Lubbock, TX United States; 2 Department of Psychology University of California at Berkeley Berkeley, CA United States; 3 Department of Psychiatry and Human Behavior Brown University Providence, RI United States

**Keywords:** ecological momentary assessment, suicidal ideation, suicidal behavior, actigraphy, sleep, cognitive control, longitudinal, affect, impulsivity

## Abstract

**Background:**

US suicide rates have risen steadily in the past decade, and suicide risk is especially high in the months after discharge from inpatient psychiatric treatment. However, suicide research has lagged in examining *dynamic within-person processes* that contribute to risk over time among individuals known to be at high risk of suicide. Almost no research has examined how affective, cognitive, and physiological processes change over minutes, hours, or days to confer risk of suicidal behavior in daily life.

**Objective:**

This protocol describes a longitudinal study designed to examine real-world changes in risk of suicide across multiple assessment domains. Specifically, the study involves following adults known to be at high risk of suicide after discharge from inpatient psychiatric care using self-report, interview, actigraphy, and behavioral methods to identify proximal contributors to suicidal thoughts and behaviors. First, we hypothesize that negative affective experiences, which are featured in most major suicide theories, will comprise a latent factor indicative of psychache (emotional pain), which will predict increases in suicidal thinking over time. Second, we hypothesize that poor inhibitory control in the context of negative affective stimuli, as well as emotion-related impulsivity, will predict the transition from suicidal thinking to suicidal behavior over time. Third, we hypothesize that short sleep duration will precede within-person increases in suicidal ideation as well as increased odds of suicidal behavior among those reporting suicidal thoughts.

**Methods:**

The desired sample size is 130 adults with past-week suicidal thoughts or behaviors who are receiving inpatient psychiatric treatment. Participants will complete a battery of measures while on the inpatient unit to assess negative affective experiences, emotion-related impulsivity, inhibitory control, typical sleep patterns, and relevant covariates. After discharge from inpatient care, participants will complete 4 weeks of signal-contingent ecological momentary assessment surveys, as well as mobile behavioral measures of inhibitory control, while wearing an actigraphy device that will gather objective data on sleep. Participants will complete interviews regarding suicidal thoughts and behaviors at 4 and 8 weeks after discharge.

**Results:**

The study was funded by the National Institutes of Health in November 2020. Recruitment began in April 2021. Data analysis will begin after completion of data collection.

**Conclusions:**

This study will elucidate how affective, cognitive, and physiological risk factors contribute (or do not contribute) to within-person fluctuations in suicide risk in daily life, with important implications for extant theories of suicide. Of import, the examined risk factors are all modifiable; thus, the results will inform identification of key targets for just-in-time, flexible, personalized, digital interventions that can be used to decrease emotional distress and prevent suicide among those at highest risk.

**International Registered Report Identifier (IRRID):**

DERR1-10.2196/38582

## Introduction

### Background

US suicide rates have risen steadily in the past decade [1], particularly in rural areas [2,3]. Risk of death by suicide is especially high in the months after discharge from inpatient psychiatric treatment [4,5], particularly for people hospitalized because of suicidal thoughts and behaviors (STB) [6]. Despite this well-documented risk, research has made little progress in improving suicide prediction over the past 50 years [7]. Suicide research has been stymied by a focus on between-person factors that differentiate those who have and have not attempted (or died by) suicide, but many of these risk factors are relatively static, such as gender and race; research has lagged in examining dynamic, within-person processes that contribute to shifting risk over time among individuals who are already known to be at highest risk [8].

Leading *ideation to action* theories have enumerated negative affective experiences thought to contribute to suicidal ideation (SI), such as low connection [9], pain [9,10], hopelessness [9,11], thwarted belongingness [12], burdensomeness [12], defeat [13], and entrapment [13]. Negative affective experiences have been associated with between-person differences in current and past SI [9,12,13], and there is some evidence that within-person fluctuations in negative affective experiences may proximally predict SI [14-16]. Risk factors that are associated with the transition from SI to suicide attempts (SA) have been far less well studied [17]. More broadly, almost no work has examined our hypothesized cognitive, behavioral, and physiological risk factors of SA in daily life. We describe our protocol for a novel, transtheoretical, comprehensive examination of near-term risk factors for SI and SA among adults leaving psychiatric inpatient care, using multimodal ambulatory assessment of affective, cognitive, and physiological processes in an intensive longitudinal design.

### Negative Affective Experiences

Shneidman [10] argued that suicide is fundamentally rooted in unbearable psychache, “hurt, anguish, soreness, aching, psychological pain.” Psychological pain is robustly associated with suicide, whether because of psychiatric disorders [18], stressful life events [19], or physical pain or illness [20,21]. Nonetheless, modern suicide theories differ in the relative importance of negative affective experiences as they relate to suicide. For example, the Interpersonal-Psychological Theory [12,22] focuses on interpersonal facets of pain, namely perceived burdensomeness and thwarted belongingness [23]. The Three-Step Theory formulated by Klonsky [9] takes a broader view of pain as a prerequisite for SI, positing connectedness as a protective factor against severe SI in the context of pain. Both the Three-Step Theory and the hopelessness model described by Beck [11] emphasize hopelessness regarding the likelihood of negative affective experiences improving over time as a key predictor of SI. The Integrated Motivational-Volitional model described by O’Connor [13] argues that SI is prompted by feelings of defeat (consistent with the view expressed by Baumeister [24] of suicide as escape from aversive self-awareness) and entrapment.

Although these models indicate multiple nuanced facets of negative affective experiences, it is unclear whether people experiencing suicidality differentiate these facets and whether they have differential predictive power. Recent network analyses suggest that many of these negative affective experiences cluster together among individuals [25]. However, to date, these studies have not conducted analyses of how these negative affective experiences covary over time within individuals or how they differentially predict outcomes. We aim to test the coherence of negative affective experiences to determine whether these are, in fact, differentially related to SI or whether they are more accurately conceptualized as indicators of the broader construct of emotional pain (eg, psychache).

Although a plethora of cross-sectional studies have shown associations between these negative affective experiences and SI [26-33], little work is available on negative affective experiences as prospective predictors of SI. Longitudinal work has primarily examined negative affective experiences as predictors of SI over months or years [34,35] rather than as predictors of within-person short-term changes in SI. Recent ecological momentary assessment (EMA) research has demonstrated that many negative affective experiences fluctuate markedly at the within-person level, as does SI [15,36-40]; however, these studies have been limited by small sample sizes [15,36], short durations [37,38], and use of analog (community) samples [39-41]. Furthermore, negative affective experiences tell us little about which people with suicidal thoughts will go on to attempt, or die by, suicide, a central issue across ideation-to-action models.

### Emotion-Related Impulsivity

Research examining the broad domain of impulsivity in relation to STB has been mixed [41,42]. However, emotion-related impulsivity, the tendency to behave impulsively during high-arousal negative or positive affective states [43-46], shows clear associations with STB [47-55], nonsuicidal self-injury (NSSI) [56-60], and self-rated likelihood of future SA [51]. Furthermore, emotion-related impulsivity seems to amplify the association between other suicide risk factors, such as negative affective experiences, and SA [48]. Daily diary research further suggests that high emotion-related impulsivity is related to stronger within-person associations between negative affective experiences and NSSI urges [61], consistent with literature demonstrating that emotion-related impulsivity is characterized by problematic responses to negative affective experiences, rather than by greater affective intensity itself [44,45,62-66]. Emotion-related impulsivity is genetically driven and highly stable over time [67] and may thus serve as a trait-like marker of SA risk among those who experience SI.

### Cognitive Inhibition

Correlational research suggests that deficits in inhibitory control in the context of negative affective experiences are associated with SA and NSSI [47,60,62,68-70], especially among psychiatric populations [49]. Null effects for inhibitory control deficits related to SA have emerged when there was a lack of consideration of affective state [69-71]. That is, meta-analyses indicate that poor inhibitory control is tied to STB when people are tested while experiencing depressed mood or major depressive disorder or when negative stimuli are incorporated into the task [53,72]. This pattern of results highlights the need for integration of affective and cognitive domains jointly to consider STB risk [46,49,62,73].

### Sleep Disturbances

Sleep disturbances are associated cross-sectionally with SI and SA [74-77], and sleep problems predict later SA in adolescents [78]. Most of this work has used self-report omnibus measures of sleep [75], with limited ability to identify specific sleep domains as they relate to STB or to disentangle effects of objective and subjective sleep disturbances. To our knowledge, only 2 studies have examined prospective associations between actigraphy-assessed sleep and subsequent SI: Littlewood et al [79] found that short sleep duration predicted within-person changes in SI across days, and Bernert et al [80] found that variability in sleep duration predicted later SI above and beyond depression. No published work has examined objective sleep indices as predictors of SA in daily life.

However, there is strong evidence from neuroimaging [81] and behavioral research using naturalistic [82] and experimental [83] paradigms that sleep disturbances diminish inhibitory control [84-87] and that sleep problems are associated with other self-harm behaviors that can occur impulsively, such as NSSI [88]. Furthermore, prior work has shown that sleep disturbances contribute to impulsivity among patients diagnosed with psychiatric disorders [88]. Thus, based on the limited literature, we predict that reduced total sleep time (compared with an individual’s mean sleep duration) will affect likelihood of SA in the context of SI through decays in inhibitory control.

### Hypotheses

The goal of this study is to integrate examination of affective, cognitive, and physiological risk factors for short-term changes in suicidal thinking and suicidal behaviors among adults known to be at high risk within an ideation-to-action framework. To that end, our hypotheses are as follows:

- Hypothesis 1a (H1a): within-person increases in affective suicide risk factors conceptually related to psychache (negative affective experiences) will individually precede within-person increases in SI.
- Hypothesis 1b (H1b): negative affective experiences will co-occur in daily life such that they are best conceptualized as indices of a single latent construct capturing emotional distress (psychache), which will itself predict short-term increases in SI.
- Hypothesis 2a (H2a): among those with SI, higher baseline self-reported emotion-related impulsivity will predict greater odds of SA over follow-up.
- Hypothesis 2b (H2b): among those with SI, poorer baseline behavioral indices of inhibitory control in the context of negative affective stimuli will predict greater odds of SA over follow-up. We also predict that, among those with current SI during EMA, lower EMA-assessed inhibitory control will prospectively predict subsequent SA.
- Hypothesis 3a (H3a): during the EMA protocol, short sleep duration will predict within-person increases in SI.
- Hypothesis 3b (H3b): during the EMA protocol, short sleep duration will predict greater risk of next-day SA among those with SI.
- Exploratory hypothesis 3c (H3c): the within-person effects of short sleep duration on SA will be mediated by within-person changes in inhibitory control assessed during EMA.

**Figure 1:**
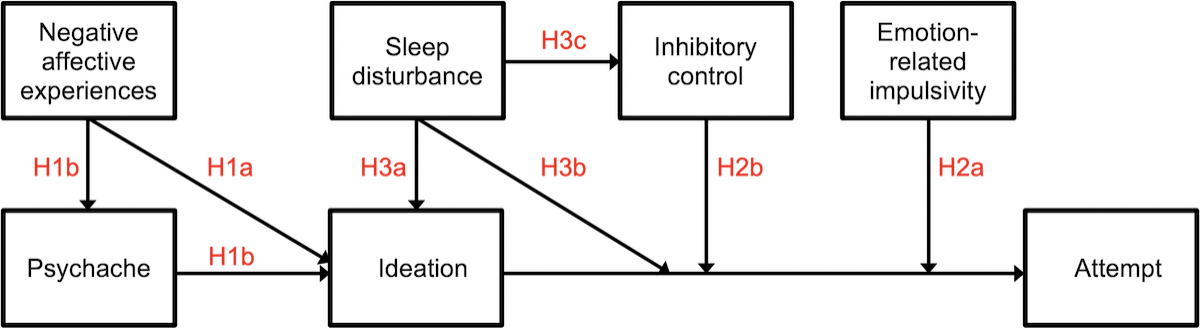
Visual depiction of study hypotheses.

A visual depiction of these hypotheses is provided in Figure 1.

## Methods

### Project Overview

This is a single-group, repeated-measures (within-persons) observational study following adults with recent STB receiving inpatient psychiatric treatment (N=130). An ethnically diverse sample of participants will be recruited during inpatient psychiatric treatment in Lubbock, Texas, United States, based on past week STB. Eligible and interested participants will then complete the informed consent process, self-report surveys, clinical interviews, a computer task assessing cognitive inhibition, and a debriefing procedure. After discharge from inpatient care, participants will engage in a 4-week (28-day) EMA protocol that involves receipt of 7 signal-contingent self-report survey prompts per day between participants’ self-reported wake times and bedtimes. Participants will also be prompted to complete an abbreviated version of the cognitive inhibitory control task after each survey and asked to wear an actigraphy device for the 28-day follow-up period to assess objective indicators of sleep. Participants will be invited to complete a remote (phone or videoconference) follow-up interview assessing recent STB at 4 weeks and 8 weeks after discharge.

### Ethics Approval

All study procedures have been approved by the Texas Tech University Institutional Review Board (IRB2020-713; October 27, 2020). The study procedures were further approved by the first recruitment site (Covenant Health) on March 15, 2021. The study procedures, hypotheses, and all relevant documents were preregistered on the Open Science Framework on August 25, 2021 [89].

### Power Analyses and Sample Size Considerations

Given variability in EMA adherence, we conducted power analyses for 100 participants, while planning to recruit 130 participants to account for those with inadequate compliance. Most study aims will be tested using dynamic structural equation modeling, as described herein. In a recent Monte Carlo simulation study [90], adequate power (>0.80) for modeling of a single process (eg, SI) was demonstrated for moderate effects where n=100 with as few as 50 repeated measures (*t*=50). Thus, this study (*t*=196, 7 prompts per day for 28 days) is well powered even with a compliance rate as low as 57.1% (112/196; 4 prompts per day for 28 days) to examine within-person processes (H1a, H1b, and H2b). To provide more conservative estimates of power, a series of simulations with 100 replications were conducted for a random-effects (multilevel) logistic outcome using R software (The R Foundation for Statistical Computing) [91]. The assumptions for these simulations were as follows: (1) n=100, (2) within-person effects for all predictors, (3) standard normal distributions for all predictors, and (4) an intercept variance of 0.3. On the basis of these models, the study is well powered (>0.8) to detect standardized within-person effects as low as 0.1 with compliance as low as 64%.

For H2a, we used G*Power (Heinrich Heine University) [92] to conservatively determine the minimum detectable effect predicting any SA over follow-up with n=100, Cronbach α=.05, and β=.8, controlling for a moderate effect of SI on SA (0.3). We assumed an event proportion of 10% for the outcome based on recent literature [93]. On this basis, the study is powered to detect an odds ratio >2.63 for the effect of baseline emotion-related impulsivity or inhibitory control on follow-up SA risk. By way of comparison, recent research has demonstrated significant predictors of follow-up SA risk with odds ratios from 6 [94] to 13 [93].

For H3a and H3b, the models are identical to those for H1a, H1b, and H2B, except that analyses are conducted at the day level (*t*=28) to examine the effects of sleep. Previous studies have shown significant effects for the impact of sleep quality on next day SI (β=–.11) [79]. Although examination of within-day fluctuations in inhibitory control is exploratory (H3c), prior work showing large differences in inhibitory control between self-harm and control groups (Cohen *d*=0.84) [70] allowed us to conservatively estimate how day-level inhibitory control predicts SI and SA. Thus, we repeated our simulations outlined for H1a using *t*=28 and single predictors for sleep (H3a and H3b) and inhibitory control (H3c) in separate models. Models converged across repetitions, indicating that with n=100, the study is well powered (>0.8) to find standardized regression effects as low as 0.15 (representing small effects) for the within-person regressions of SI and SA on day-level inhibitory control or sleep disturbance.

### Study Procedures

#### Recruitment and Informed Consent

**Figure 2:**
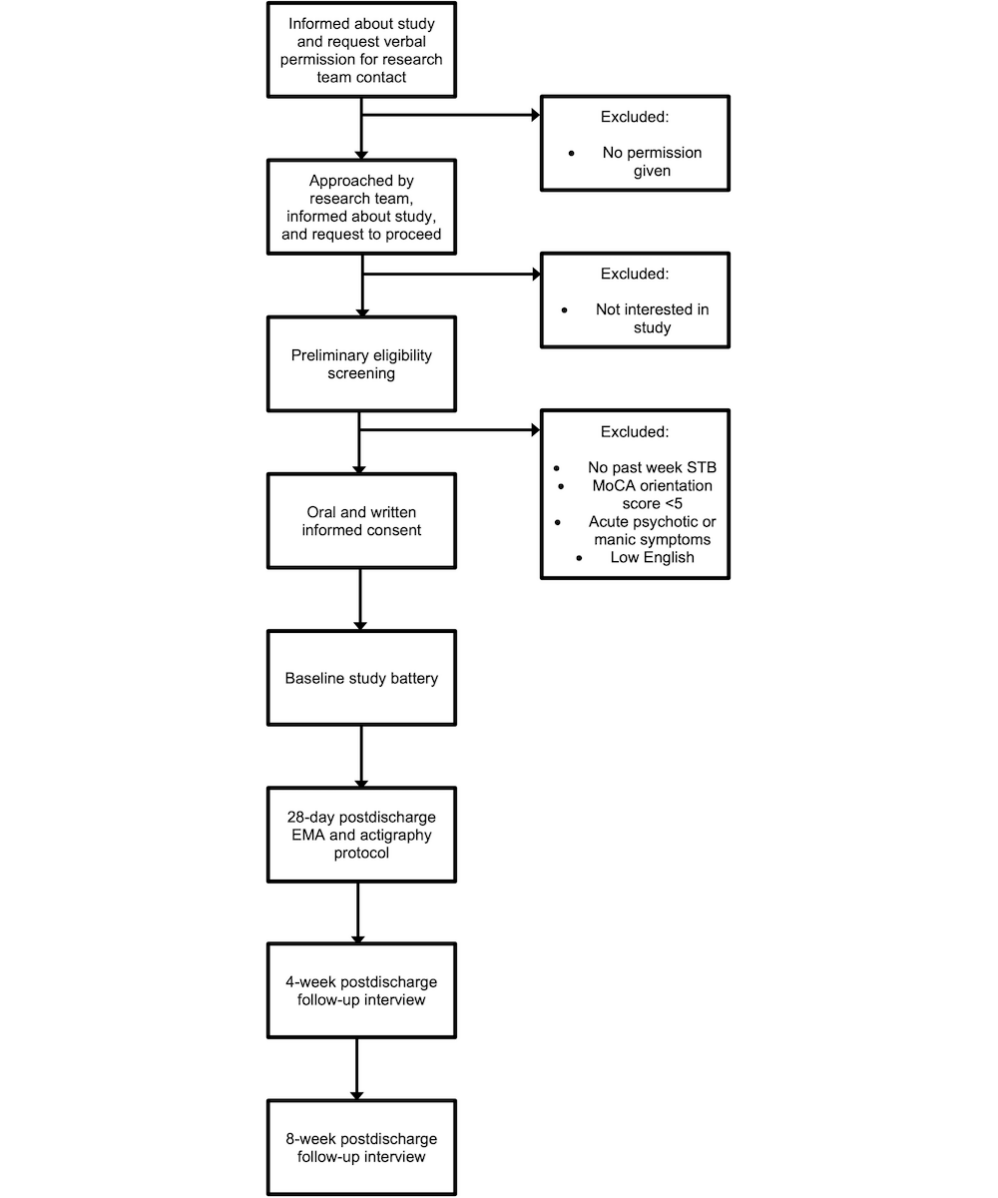
Study recruitment, enrollment, and procedures flow chart. EMA: ecological momentary assessment; MoCA: Montreal Cognitive Assessment; STB: suicidal thoughts and behaviors.

Participants will be recruited from the inpatient behavioral health units located in Lubbock, Texas. The inclusion criteria are as follows: (1) current inpatient behavioral health treatment, (2) aged at least 18 years (no maximum age), and (3) SI or suicidal behavior in the week before admission. The exclusion criteria are as follows: inability to complete tasks because of (1) impaired mental status (orientation score <5 on the Montreal Cognitive Assessment), (2) acute psychotic or manic symptoms, or (3) low English fluency. Treatment team members (eg, attending physicians and nursing staff) will identify individuals who are potentially eligible for the study and ask these individuals for verbal consent to be approached by a team member regarding the study. Informational flyers describing the study will be provided to treatment team members to offer to interested patients; team members will also be provided scripts to describe the study orally. With approval, a study team member will then approach potential participants during times that do not conflict with treatment or unit activities to discuss the study. The study team member will meet with the potential participant in a private area on the unit to explain the study design, procedures, risks and benefits, compensation, confidentiality and its limits, and eligibility criteria. Oral and written consent will be obtained from participants before proceeding. Study consent forms, the prescreening form, and summary information sheets are available alongside the Open Science Framework preregistration [89]. A diagram showing the flow of potential participants through study procedures is presented in Figure 2.

#### Baseline Session

The baseline session will take place on the inpatient behavioral health unit and last for 2 to 3 hours. Participants will complete self-report surveys, clinical interviews, a computer task (Emotional Stop-Signal Task), and a debriefing procedure. Participants will also be instructed on how to complete the follow-up EMA surveys and mobile computer tasks, as well as how to use the actigraphy device. A complete list of self-report measures and clinical interview assessments is provided in Table 1. A summary of study data collection procedures as well as all baseline measures are provided alongside the Open Science Framework preregistration [89].

**Table 1:** Self-report and clinical interview measures for the baseline session.

Measure type and construct			Measure
**Self-report**			
	Age, race, ethnicity, gender, and other demographics	Demographics	
	Positive and negative affect	Positive and Negative Affect Schedule	
	Emotion-related impulsivity	Three-Factor Impulsivity Scale	
	Thwarted belongingness	Interpersonal Needs Questionnaire	
	Perceived burdensomeness	Interpersonal Needs Questionnaire	
	Defeat	Defeat Scale	
	Entrapment	Entrapment Scale	
	Hopelessness	Beck Hopelessness Scale (4-item version)	
	Emotional pain	Psychache Scale	
	Physical pain interference	PROMIS^a^ Pain Interference Scale	
	Sleep chronotype	Smith Morningness-Eveningness Scale	
	Severity of insomnia	Insomnia Severity Index	
	Symptoms of sleep disorders	Sleep Disorders Symptoms Checklist-25	
	Coping strategies for suicidality	Suicide-Related Coping Scale	
	Exposure to traumatic events	List of Threatening Experiences Questionnaire	
	Recent depression, anxiety, and stress symptoms	Depression Anxiety Stress Scales (21-item version)	
	Capability for suicide	ACSS^b^ Fearlessness About Death Subscale	
	Access to lethal suicide means	PhenX^c^ Access to Lethal Means protocol	
	Onset and typical patterns of menstrual cycle	Menstrual history (women only)	
**Interview**			
	Past week suicidal ideation or behavior	Past Week Suicide Assessment	
	Lifetime, past year, past month, and past week suicidal ideation and behavior	Columbia Suicide Severity Rating Scale	
	Lifetime, past year, past month, and past week nonsuicidal self-injury urges and behaviors	Self-Injurious Thoughts and Behaviors Interview (NSSI^d^ items)	
	Lifetime and past year alcohol use disorder symptoms	Structured Clinical Interview for DSM-5^e^ Disorders (Alcohol Use Disorder items)	
	Lifetime and past year substance use disorder symptoms	Structured Clinical Interview for DSM-5 Disorders (Substance Use Disorder items)	
	Borderline personality disorder symptoms	Structured Interview for DSM-IV^f^ Personality Disorder (Borderline Personality Disorder items)	
	Current medications and dosages	Medication Interview	

^a^PROMIS: Patient-Reported Outcomes Measurement Information System.

^b^ACSS: Acquired Capability for Suicide Scale.

^c^PhenX: Phenotypes and Exposures.

^d^NSSI: nonsuicidal self-injury.

^e^DSM-5: Diagnostic and Statistical Manual of Mental Disorders, Fifth Edition.

^f^DSM-IV: Diagnostic and Statistical Manual of Mental Disorders, Fourth Edition.

Participants will complete an adapted version of the Emotional Stop-Signal Task [95] to behaviorally assess impulsivity. To focus on emotion-relevant response inhibition, we use an adapted version in which participants are presented with either neutral or negatively valenced images. In *Go* trials (75%), participants press a key to indicate the valence of the image within 1500 milliseconds. *Stop* trials (25%) involve a sound played through headphones at a variable delay after stimulus presentation and indicate that participants should inhibit their response to the image. The delay begins at 250 milliseconds and then increases or decreases by 50 milliseconds in response to successful or failed inhibitory control, respectively. The scores, then, reflect the difference in commission error rates between negative and neutral stimuli, which is an indicator of deficits in cognitive inhibition specific to negative affective contexts. During the baseline session, participants will complete practice trials followed by 3 blocks of 64 trials (192 trials, 48 *stop* trials).

#### Intensive Longitudinal Protocol

During the baseline session, participants will be asked to provide their typical wake times and bedtimes, as well as any specific windows of time in which they will be unavailable to answer surveys (for instance, work shifts in which they are not allowed to access their phone), which are then used to schedule the EMA survey distribution. Participants who do not own a mobile phone capable of accessing the internet will be provided with a *loaner* phone to use for the EMA surveys; other participants will use their own mobile phone. All participants will be walked through practice EMA items to familiarize them with the structure of the EMA surveys and the mobile version of the Emotional Stop-Signal Task before discharge.

The EMA surveys will be distributed using a proprietary distribution system that integrates with Amazon Web Services’ Simple Notification Service system to send SMS text messaging notifications of survey availability. These notifications will include a link to a Qualtrics survey that will be available for 30 minutes only after each notification.

Participants will receive 7 pseudorandom notifications between specified wake times and bedtimes, avoiding the blackout windows specified by the participant. We will add 15 minutes to the wake time to avoid missed surveys because of variability in wake times as well as to ensure that participants will be able to respond effectively to items regarding affect and related constructs after waking. Notifications will be randomized by *binning* the participants’ available hours into 7 equal windows and then randomly choosing a survey time within each bin. Notifications cannot occur within 30 minutes of another notification. If participants do not click the survey link within 10 minutes of notification, they will receive a reminder message. At each EMA prompt, participants will be asked to complete self-report items assessed using Qualtrics and then to complete the Emotional Stop-Signal Task on their mobile phone using the Inquisit web platform (Millisecond Software, LLC; available for both iOS and Android platforms). This adapted version of the task also uses negative and neutral stimuli. At the first survey completed each day, the task will have 128 trials (64 negative and 64 neutral trials interspersed). At each subsequent pseudorandom prompt, participants will complete 64 trials (32 negative and 32 neutral trials interspersed) to reduce participant burden while allowing within-person dynamic modeling of changes in affective inhibitory control. The domains assessed using EMA self-report items are listed in Table 2, and specific items are provided alongside the Open Science Framework preregistration [89].

**Table 2:** Domains assessed using self-report ecological momentary assessment items.

Survey and construct		Number of items
**Morning survey only**		
	Sleep quality	1
	Sleep characteristics (time to bed, time to wake up, awakenings, and nightmares)	8
	Menstrual cycle (only for participants with regular menstrual cycle reported at baseline)	2
**All surveys**		
	Current suicidal ideation	2
	Current urges for nonsuicidal self-injury	2
	Current negative affect	6
	Current positive affect	2
	Current hopelessness	1
	Current agitation	2
	Current emotional pain	2
	Current defeat	2
	Current entrapment	2
	Current self-criticism	2
	Current suicide capability	4
	Current physical pain interference	1
	Current belongingness	2
	Current burdensomeness	2
	Current perceived emotional support	2
	Current perceived practical support	2
	Since last survey: suicidal behavior	1
	Since last survey: nonsuicidal self-injury	1
	Since last survey: stressors	3
	Since last survey: complimented or praised	1
	Since last survey: alcohol use	2
	Since last survey: drug use	4

At baseline, participants will also be provided with the actigraphy device (wActiSleep-BT; ActiGraph, LLC) and instructed on how to wear it during the follow-up period. Participants will be asked to wear the device as much as possible for 28 days after discharge. The device continuously records activity through accelerometer (approximately 30-hertz data sampling with timestamps) with a single charge typically exceeding 28 days [96]; however, participants will also be provided with a charger and asked to charge the device at least twice during follow-up at times when the watch would not otherwise be worn, such as when bathing. The device continuously records data to provide valid scores for a variety of indices, including sleep duration, sleep onset latency, total awakenings, length of awakenings, overall sleep efficiency, intensity of movement during sleep awakenings, and light exposure during sleep [97]. Participants will be provided with a self-addressed, postage-paid envelope to return the actigraphy device, charger, and loaner phone (if applicable) to the research team after study completion.

#### Follow-up Interviews

Participants will be asked to complete a brief remote (phone or videoconference) follow-up interview assessing STB at 4 weeks and 8 weeks after discharge, with the goal of ensuring that the follow-up data capture all experienced STBs even if not reported on the EMA surveys. Specific follow-up interview items are provided alongside the Open Science Framework preregistration [89].

#### Risk Assessment, Crisis Response Planning, and Strategies for Retention and Study Engagement

Participants who consent to participate in the EMA portion of the study will be contacted on a regular basis to identify concerns with the study, issues regarding receipt of the EMA notifications, and to assess ongoing suicide risk. All participants, regardless of their level of compliance with the EMA protocol and indicated suicide risk, will be contacted by the research team through SMS text messaging weekly (4 times over the 28-day period). Participants will also be contacted on day 5 of the EMA protocol if under half of the expected surveys are completed during the first 4 days of the protocol to remind them of the compensation rate per survey and to ascertain if any issues were inhibiting completion of the surveys. Participants will then be contacted if they demonstrate 1, 2, and 4 consecutive days of nonresponse to any surveys. After the first occurrence of extended periods of noncompletion, check-ins will only be conducted after 2 or 4 full days without completing any surveys to avoid excessive or intrusive follow-ups.

Participants will also be contacted within 24 hours of any EMA surveys in which they indicated engaging in suicidal behavior. These contacts will follow a specified risk assessment and crisis response plan dependent on participants’ responses and indicated level of imminent risk. The procedures used during these follow-ups are available in documentation alongside the Open Science Framework preregistration [89].

#### Compensation

Participants’ compensation will depend on the specific study components completed. Payments will be provided either through a digital Amazon gift card (by email) or a physical Walmart gift card (during face-to-face interactions or by mail). All participants who complete the informed consent process at baseline will receive a US $30 gift card, regardless of how many measures they complete.

All participants who consent to complete the EMA and actigraphy follow-up portion of the study will receive a US $50 gift card, regardless of the number of surveys completed or amount of time the actigraphy device is worn. Participants will receive an additional US $1 for each survey completed (up to an additional US $196). If participants complete at least 75% (147/196) of the surveys received, they will receive a bonus US $25 gift card. Participants will receive a US $20 gift card for completion of a follow-up interview, up to a maximum of US $40 for both the 4-week and 8-week follow-up assessments.

#### Data Analysis Plan

Before conducting analyses, we will clean and check data for errors, address outliers, ensure that statistical assumptions for analyses have been met, and examine potential confounders to include in subsequent analyses as warranted. Variables that may exhibit skewness (eg, number of lifetime episodes of NSSI) will be examined for skewness and kurtosis and, if necessary, either rank transformed or log transformed. We will include the effect of time (196 prompts over 28 days) to account for habituation and examine potential nonlinear effects of, for instance, age. Most analyses will use time-series (dynamic) structural equation modeling [98,99], which uses Bayesian estimation, conducted with MPlus statistical software (Muthén & Muthén) [99]. Dynamic structural equation modeling is robust with regard to high rates of missing data, zero-inflated categorical variables, and variable time intervals among observations, all of which are common in EMA with these types of data, and allows for latent decomposition of constructs into their constituent between- and within-person components [98]. Dynamic structural equation modeling also facilitates within-person lagged analyses to examine prospective relationships between time *t* predictors and time *t*+1 outcomes; by controlling for autoregressive effects over time, these methods can model within-person change in constructs over time.

For H1a, we will examine whether within-person changes in negative affective experiences precede proximal within-person changes in SI using 2-level random slope models. Each of 7 momentary negative affective experiences reported at time *t* will be regressed on itself at time *t–*1, producing a measure of residualized change in each negative affective experience. Momentary SI reported at time *t* will be regressed on itself at time *t–*1, as well as the 7 negative affective experience change indicators, yielding an estimate of the within-person effect of changes in all negative affective experiences on subsequent within-person changes in SI, controlling for autoregression. For H1b, we will examine the within- and between-person factor structures of the 7 negative affective experiences using multilevel exploratory factor analysis [100]. Using established best practices, we will identify the most appropriate structure and use these within-person factors to predict changes in SI over time, similar to the procedures used for H1a.

For H2a, we will estimate the effects of baseline emotion-related impulsivity and inhibitory control on odds of SA over follow-up, controlling for between-person differences in SI severity. To do this, person-mean estimates of SI and person-maximum estimates of SA (yes or no) will be calculated using EMA data. SA can then be predicted by baseline emotion-related impulsivity and inhibitory control scores using logistic regression analysis, controlling for participants’ SI severity. For H2b, we will then test whether within-person changes in state inhibitory control precede subsequent SA among individuals reporting SI. Using a 2-level random slope model, we will model time *t* inhibitory control (controlling for inhibitory control measured at time *t–*1) as a predictor of time *t*+1 SA, controlling for time *t* SI.

For sleep-related hypotheses, we will use 2-level random slope models, as described previously, but in which survey-level data on SI and SA have been pooled to create day-level variables (because sleep varies at the daily, but not survey, level). For H3a, we will use person-mean–centered total sleep time on day *d–*1 to predict next-day (day *d*) SI, controlling for day *d–*1 SI. Models for H3b will parallel those used to test H2a, whereby total sleep time on day *d*-1 will be used to predict next-day SA (day *d*), controlling for day *d* SI. For H3c, we will test whether the sleep-SA association (H3b) is mediated by within-person changes in inhibitory control. Building on the H3b model, we will add in the effect of sleep on day *d–*1 predicting inhibitory control on day *d*. To ensure that we are capturing sleep-related changes in inhibitory control, inhibitory control on day *d* will also be regressed on inhibitory control measured on day *d–*1. Finally, day *d* inhibitory control will be used to predict day *d* SA, controlling for day *d* SI. Indirect and direct effects will be calculated and tested for significance using the MPlus *model indirect* command.

With respect to the criteria used for statistical inferences, these will vary by model and hypothesis. For logistic regression analyses (H2a), 2-tailed *P* values <.05 will be used as our criteria to make inferences. For dynamic structural equation models (H1a, H1b, H2b, H3a, H3b, and H3c), which use Bayesian estimation, 95% credibility intervals will be used; those intervals that do not include 0 will be evidence of a statistically significant effect. For multilevel exploratory factor analysis (H1b), we will examine unrestricted models with 1 to 7 factors at the within- and between-person levels, consistent with the components of the negative affective experiences construct. We will determine the most appropriate model fit based on an examination of model fit indices [101] as well as eigenvalues.

## Results

This study was funded by the National Institute of Mental Health (R21 MH124794) in November 2020 with a start date of December 1, 2020, and an end date of October 31, 2022 (Multimedia Appendix 1). Recruitment and data collection began in April 2021, and 46 individuals were consented into the study through November 2021. Of note, the first study recruitment site (Covenant Health’s adult behavioral health inpatient unit) was unexpectedly closed on November 15, 2021, which resulted in a pause in recruitment while an alternative site was identified. As of February 2022, a new recruitment site has provided preliminary approval to restart study data collection, which began in June 2022. Data collection is anticipated to be completed by winter 2023.

## Discussion

### Expected Implications and Conclusions

Although suicide research has proliferated in the past 50 years, this work has not moved the needle substantively on suicide prevention, primarily because of a focus on static, between-person risk factors (*who* is at risk) rather than modifiable, within-person risk factors (*when* or *why* is a particular person at risk). Major suicide theories have proposed key drivers of STB, but very little research has directly compared the relevance of constructs across theories or tested whether these risk factors are related to dynamic fluctuations in suicide risk. In addition, empirical research on hypothesized cognitive and physiological contributors to suicide risk has been limited by an inability to measure these phenomena as they occur and change in daily life.

This study aims to address limitations in the research on SI and behavior in several ways. First, by examining between- and within-person experiences of a multitude of conceptually derived negative affective experiences, the results will clarify whether these are, in fact, uniquely and differentially related to suicide outcomes or whether they are more appropriately construed as indicators of a single latent construct, such as psychache. The results will thus clarify the utility of existing theories in which fine-grained, nuanced distinctions among risk factors may (or may not) map onto experiences that people with STB themselves discriminate among in daily life. Second, by using baseline and ambulatory measures of sleep disturbance and impaired inhibitory control, the results will clarify whether these serve as more stable or trait-like indicators of risk or whether fluctuations in these experiences relate to changes in risk over time. The results will inform the development of interventions designed to modify these risk factors in their most relevant contexts to decrease the odds of suicide among those at high risk.

### Methodological and Other Considerations

The primary methodological decisions in study design were with respect to the inclusion criteria, intensity of follow-up data collection methods, and level of monitoring and responsivity to indicators of suicide risk during follow-up.

First, we considered whether to limit the sample further (eg, individuals with recent SA) or whether to expand the inclusion criteria to include anyone receiving inpatient psychiatric care, regardless of recent STB. Individuals with a history of SA are at higher risk of later suicidal behavior than individuals with SI only [102], which would increase the odds of suicidal behavior during the follow-up period and plausibly increase statistical power. However, we felt that further restriction of the inclusion criteria would be likely to slow the speed of recruitment, a significant concern for a grant-funded study with a 2-year timeline. Recruiting a more heterogeneous sample will also allow us to examine the generalizability of predictors across a more diverse patient population. By contrast, expanding eligibility to all individuals receiving inpatient psychiatric care would likely reduce the odds of suicidal behavior over follow-up compared with individuals with recent STB [4].

Second, we considered the significant trade-offs between level of detail and frequency of follow-up assessments with the need to reduce participant burden (and, relatedly, the odds of attrition from the study). Prior research has shown that a greater number of surveys per day [103] and a greater number of items per survey [104] predict lower compliance with momentary assessment (EMA) research, which then affects the ability to adequately capture outcomes of interest (STB) during the EMA period. In addition, asking follow-up questions about the nuances of STB based upon *yes* responses during the EMA may increase the odds of participants choosing to deny these experiences that do occur to avoid additional follow-up questions, which take time and may be sensitive in nature. The duration and frequency of assessments chosen for this study were based on prior research suggesting that these parameters were feasible for the population of interest [105]. Finally, the 4- and 8-week follow-up interviews were added to the study protocol in response to grant reviewer feedback as a check against missed STB that were not captured during the EMA to increase statistical power for between-person models.

Finally, intensive longitudinal studies examining suicide risk and related constructs vary in the strategies used to monitor incoming data and to respond to indicators of elevated suicide risk [106]. These decisions often involve trade-offs of study investigator and staff time, guarantees of anonymity for participants, balancing participant safety with the risks involved in interventions by outside parties, avoidance of unintentional reinforcement of STB through study staff contact, and the goals of observational versus intervention-based research. Although we considered real-time monitoring of participants’ full range of EMA responses concerning suicide risk factors and contacting participants on the basis of these responses, we ultimately determined that this was scientifically unjustified because we do not know precisely what level of SI or other risk factors are related to imminent (proximal) risk for suicide. Follow-up with participants during the EMA protocol was limited to disclosures of suicidal *behaviors* only, following the aforementioned procedures.

However, we did make several choices across the study to promote truthful responding on suicide risk indicators by participants to ensure that they had adequate access to safety planning and other crisis resources and to avoid development of an intervention study when the study aims were exclusively observational. First, participants will be informed repeatedly at baseline that the study involves many assessments of STB and that many of these experiences will not lead to violations of their confidentiality. Participants will be informed that the confidentiality of information provided during the study will only be broken if the participant indicates imminent (within 1-2 days) desire, intent, and plans to attempt suicide and the research team member is not able to develop a safety plan with the participant to address this risk. Second, crisis hotline information and local, national, and web-based resources related to self-harm and suicide will be provided to participants at the baseline session, at which time all participants will be offered the opportunity to complete a suicide safety plan with the research team; if a safety plan is created, it will be given to the participant to keep and a copy will be retained by the research team. Furthermore, at the start of each EMA survey, participants will be offered live links to call or text 24/7 crisis hotlines, alongside a reminder that responses will not be monitored in real time; the same message will be displayed on the final page of each EMA survey. Each intervening page of questions will have a link at the bottom of the page that opens a full list of resources in a separate web browser if needed. Thus, participation in the research study will involve greater-than-usual exposure to, and reminders of, available crisis supports but will not involve routine additional intervention by the research team based on SI or related experiences.

### Strengths and Limitations

As with all empirical research, this study includes several important limitations to consider. Several of these factors relate to the nature of the specific sample being recruited for the project. First, because of the nature of recruitment from an inpatient psychiatric treatment setting, the study sample is geographically restricted to adults from Lubbock, Texas, and the surrounding areas, and the results may differ from those obtained in larger metropolitan areas and in other parts of the United States, as well as internationally. Second, patients with manic or psychotic symptoms as well as those with cognitive impairment may be underrepresented in the sample, given the exclusion criteria related to the ability to provide informed consent to participate in the study. Third, the decision to base eligibility on recent STB will necessarily limit our ability to make inferences about postdischarge suicide risk among patients with no, or more distal, history of STB, who are at lessened, but nonzero, risk of postdischarge STB [4,5]. Fourth, although adequately powered, the desired sample size (N=130) will limit the ability to examine potential moderators of the study results across demographic and clinical characteristics (eg, race and ethnicity, sexual orientation, and mental health diagnoses).

Limitations also exist with respect to study methodology. Owing to concerns regarding the length and intensity of our baseline session, many study constructs will be assessed using self-report survey instruments, which can be subject to response biases and whose associations with each other may be attributable to shared method variance. Although several strategies have been implemented to improve completion rates of self-report, behavioral, and physiological data collection during the EMA protocol, we expect participants to vary in level of adherence to study procedures, which will also influence data quality and statistical power for planned analyses.

Despite these limitations, the study has numerous strengths. Data will be collected from a population known to be at exceptionally high risk for STB and for whom prior research has been limited by practical challenges involved in collecting data from inpatient psychiatric settings. This particular sample will be drawn from a high-need region where mental health care access is limited, facilitating understanding of risk factors for suicide in an underserved community where mobile interventions may be particularly critical to ameliorate suicide risk. Using a transtheoretical approach to understanding the transition from suicidal thoughts to behaviors, this study will facilitate direct comparison of key hypothesized risk factors as potential real-world contributors to suicide, moving forward both theoretical and empirical understanding of these phenomena. Furthermore, data collection will span a number of methodological approaches, including self-reports, interviewer ratings, behavioral measures, and ambulatory physiological readings, facilitating multi-trait, multi-method examination of STB risk. Finally, and most importantly, the intensive longitudinal design provides a novel opportunity to understand within-person changes in STB risk over time in contrast to prior work that has focused on between-person characteristics that relate to historical experiences of STB.

### Dissemination Plan and Future Directions

Numerous steps will be taken to ensure that data collected through this study are used in an effective manner to improve the scientific understanding of STB and the experiences of people with STB interacting with the mental health system. Upon beginning data collection, the study procedures and aims were registered on the Open Science Framework [89] to improve the transparency and reproducibility of the study analyses and findings. Raw (deidentified) data from this study will also be made available to other researchers through the National Institute of Mental Health Data Archive (refer to the *Data Availability* section). Research outputs generated from this project (eg, conference presentations and scientific manuscripts) will be shared broadly with other STB researchers as well as the general public through translational mechanisms (eg, posters and social media dissemination).

Data from this study will serve as the foundation of future projects to examine tested risk factors for STB in greater detail. First, our data on negative affective experiences as contributors to suicidal thinking will inform the design of future EMA tools tailored to those experiences most strongly predictive of within-person changes in SI. These data will also clarify which therapeutic interventions may be most effective for addressing SI; for instance, if thwarted interpersonal needs are identified as key proximal SI risk factors, interpersonally focused interventions for STB may be more effective than intrapersonally focused interventions. Second, should impaired cognitive control be identified as a key factor in the transition from SI to SA, future research could test whether cognitive interventions are effective in reducing STB risk in this population. Finally, future research may build upon our sleep physiology findings by developing and testing suicide-specific sleep treatments among groups known to be at high risk, such as adults leaving inpatient psychiatric care.

## Appendix

Multimedia Appendix 1 Peer reviewer report from the National Institute of Mental Health (NIMH).

## Abbreviations

EMA: ecological momentary assessment
NSSI: nonsuicidal self-injury
SA: suicide attempts
SI: suicidal ideation
STB: suicidal thoughts and behaviors

## Data Availability

Consistent with the requirements of National Institute of Mental Health grant funding, all study data will be deidentified and uploaded to the National Institute of Mental Health Data Archive at 6-month intervals.
