# Supplementary material for: Dynamic Regulatory Processes in the Transition From Suicidal Ideation to Action in Adults Leaving Inpatient Psychiatric Care: Protocol for an Intensive Longitudinal Study
Source: JMIR Res Protoc. 2022 Jun 30;11(6):e38582. doi: 10.2196/38582 (PMC9284352; doi:10.2196/38582)
Supplement: Multimedia Appendix 1 [file resprot_v11i6e38582_app1.pdf]

SUMMARY STATEMENT

(Privileged Communication)

PROGRAM CONTACT:
Janani Prabhakar
301-827-1321
janani.prabhakar@nih.gov

Release Date: 07/27/2020
Revised Date:

Application Number: 1 R21 MH124794-01

Principal Investigator

VICTOR, SARAH ELIZABETH

Applicant Organization: TEXAS TECH UNIVERSITY

Review Group: ZMH1 ERB-S (07)
National Institute of Mental Health Special Emphasis Panel
Dysregulation and Proximal Risk for Suicide Review Meeting (R01 & R21)

Meeting Date: 07/09/2020
Council: OCT 2020
Requested Start: 12/01/2020
RFA/PA: MH20-326
PCC: A2-AID2

Project Title: Dynamic Regulatory Processes in the Transition from Suicidal Ideation to Action: The Roles of Cognitive Control, Emotion-Related Impulsivity, and Sleep in the Context of Negative Affective Experiences
SRG Action: Impact Score:35
Next Steps: Visit https://grants.nih.gov/grants/next_steps.htm
Human Subjects: 30-Human subjects involved - Certified, no SRG concerns
Animal Subjects: 10-No live vertebrate animals involved for competing appl.
Gender: 1A-Both genders, scientifically acceptable
Minority: 1A-Minorities and non-minorities, scientifically acceptable
Age: 3A-No children included, scientifically acceptable

Project Year	Direct Costs Requested	Estimated Total Cost
1	150,000	231,128
2	125,000	192,607
TOTAL	275,000	423,735

1R21MH124794-01 VICTOR, SARAH

SCIENTIFIC REVIEW OFFICER'S NOTES

RESUME AND SUMMARY OF DISCUSSION: This is an R21 application received in response to the NIMH Dysregulation and Proximal Risk for Suicide (Clinical Trial Optional) RFA that proposes to evaluate proximal predictors of within-person changes in suicidal ideation (SI) and suicide attempt (SA) across 28 days among 130 adults following inpatient hospitalizations for suicide attempts (SAs). The application includes an innovative set of methods (including ecological momentary assessment (EMA), actigraphy, and within-person assessment of multiple RDoC domains) across units of analysis. The goals of the proposed project are highly significant with regard to public health and in addressing the goals of the RFA. While not sufficiently emphasized in the application, the likely inclusion of a large portion of Latinx subjects is an important strength of the proposed study. The investigative team is led by an impressive early career PI who has assembled a team of collaborators with diverse experience in suicide research and of relevance to the proposed project. While the scientific environment appears generally supportive, there is some lack of clarity provided about the relationship between the PI/applicant institution and the recruitment site, and questions arise about the ability to recruit the proposed sample within the short proposed timeframe. Additional weaknesses are identified in the highly optimistic power analyses (especially for SA); inadequate consideration of known sex differences in self-reported precipitants of SI/SA; and inadequate inclusion of sleep expertise as reflected in the choice of outdated retrospective sleep measures, and inadequate discussion of the strengths and weaknesses of using actigraphy to measure sleep disturbance. Overall, this is a highly significant R21 application with many strengths (including the study of a population that is at very high risk for SI/SA, and the likely inclusion of a large portion of Latinx subjects), but with some weaknesses described above.

DESCRIPTION (provided by applicant): Few NIMH-funded studies have examined contributors to short-term, within-person changes in suicidal ideation and odds of suicidal behavior. We propose to use multi-modal assessments (self-report, behavior, actigraphy) to capture changes in several RDoC domains relevant to suicide risk. In an ideation-to-action framework, we differentiate acute risk factors for suicidal thoughts from those that contribute to the transition from suicidal ideation to behavior. We will test our theoretical model using a 28-day intensive longitudinal design in a sample of suicidal adults leaving inpatient behavioral health care, a population at particularly high risk for suicide. Aim 1: Examine a set of transtheoretical risk factors as proximal predictors of within-person changes in suicidal thoughts. Using ecological momentary assessment, we will gather repeated measures of seven hypothesized ideation risk factors, as well as a dimensional measure of ideation, to model lagged relationships between affective risk factors and subsequent ideation. We expect that each affective risk factor will show a positive association with subsequent increases in suicidal ideation, and that these risk factors will co-occur in daily life such that they are best conceptualized as a single latent construct capturing emotional suffering (psychache). Aim 2: Test self-report and behavioral measures of inhibitory control as risk factors for suicidal behavior. We expect that participants who, at baseline, report greater emotion-related impulsivity, and who show impaired response inhibition in a behavioral task using negative valence stimuli, will have higher odds of suicide attempt over follow-up. Using a novel mobile adaptation of an inhibitory control task during the ecological momentary assessment period, we expect that within-person decrements in inhibitory control will precede within-person increases in likelihood of suicide attempt among individuals thinking of suicide. Aim 3: Evaluate objectively measured sleep duration as a proximal risk factor for both suicidal ideation and suicidal behavior. Using wrist-worn actigraphy devices, we will examine how short sleep duration, compared to participants' average sleep, relates to next-day ideation and attempts. We expect that short sleep duration will predict within-person increases in suicidal thoughts, as well as increased likelihood of suicide attempts, controlling for suicidal ideation. Finally, we will test an exploratory hypothesis, that the sleep-suicide attempt association is explained by sleep-related decreases in inhibitory control in the

context of negative affect. Findings from this study will elucidate modifiable affective, cognitive, and physiological targets for just-in-time, mobile interventions to prevent suicide.

PUBLIC HEALTH RELEVANCE: The goal of the proposed work is to test affective, cognitive, and physiological processes as contributors to proximal risk of suicide in the daily lives of adults leaving inpatient psychiatric treatment. We will use ecological momentary assessment and actigraphy over a 28-day post-discharge follow-up to demonstrate how increases in negative affective experiences and decreases in total sleep time contribute to proximal within-person increases in suicidal ideation; further, we will show that decreased state (EMA) and trait (baseline) inhibitory control and acutely reduced total sleep time contribute to within-person transitions from suicidal thinking to suicidal behavior (e.g., suicide attempts). This work uses a novel approach that integrates RDOC constructs of negative valence, cognitive inhibition, and arousal (sleep) systems to elucidate proximal risk factors for suicide in a high-risk group of adults leaving intensive psychiatric care.

CRITIQUE 1

Significance: 3
Investigator(s): 4
Innovation: 3
Approach: 4
Environment: 5

Overall Impact:

The authors propose to evaluate proximal predictors of within-person changes in suicidal ideation (SI) across 28 days among N=130 adults following inpatient hospitalizations for suicide attempts (SAs). They will (1) use EMA to track self-reports of 7 negative affective experiences (e.g., low connection, pain, hopelessness) and “inhibitory control” (emotional Stop Signal Task; 1.7-5.7 min); (2) evaluate time-lagged associations between these variables and SI/SA (Columbia Suicide Severity Rating Scale); and (3) assess sleep disturbance using wrist-worn actigraphy to determine its relation to next-day SI/SA. EMA data will be collected 7 times per day (196 prompts total across 28 days). The authors expect that EMA-evaluated inhibitory control and response inhibition, poor “emotion-related” impulsivity (self-report), and poor sleep will predict SI and suicide attempts (SA) during the 28 day follow-up period. The proposal brings together a team of collaborators with diverse experience with suicide research, and has clear public health significance (predicting SI/SA in adulthood among those at highest risk). Innovation is strong, with between- and within-participant assessments across units of analysis as a noted strength. Assessment of sleep is also a strength. Concerns arise over poor mapping of measures into specific RDoC constructs and use of measures that combine multiple RDoC constructs into a single dimension. In addition, tests of interactions among core RDoC constructs—a goal of the RFA—are not proposed. Concerns about recruitment, failure to consider known sex differences in primary independent variables (e.g., impulsivity, response inhibition), and limited power to detect interaction effects also emerge.

1. Significance:
Strengths

- In the author’s words, “U.S. suicide rates have risen steadily in the past decade, particularly in rural areas. Risk of death by suicide is especially high in the months after discharge from inpatient psychiatric treatment.” Thus, there is no doubt the proposal, in which the authors seek to identify proximal markers of suicide risk in the 28 days following inpatient discharge among adults who attempted suicide, is of high public health significance.
- The goal of objectively quantifying ideation-to-action models is important. To date, much of the support for these models is indirect and conjectural.

- Following and characterizing a highly vulnerable group may have important implications for future treatment.

Weaknesses

- The authors do not consider sex differences in self-reported precipitants of SI/SA. Both are well documented. Men are more likely to engage in SB impulsively whereas women more often report emotion dysregulation as a precipitating mechanism. These divergent findings (which may implicate different RDoC domains by sex) are not acknowledged or considered. This suggests that (1) impulsivity and emotional lability should be separated (which may be problematic for the emotion-related impulsivity construct), and (2) sex should be included as a between-persons variable (i.e., in addition to within-persons modeling). Failure to account for sex effects detracts from significance.

2. Investigator(s):
Strengths

- The investigative team brings together a wide range of competencies, including experience with suicidal populations, EMA, and assessment of all measures chosen.
- PI Sarah Victor, Ph.D. (PI), is Assistant Professor of Psychological Sciences at Texas Tech. Her research examines suicidal thoughts and suicidal behaviors among adolescents and adults. This work includes use of daily diaries, EMA, and lab tasks. She has already published in first-tier journals with several top suicide researchers.
- Co-I Jason Van Allen, Ph.D., is Associate Professor of Psychological Sciences at Texas Tech. He is a child clinical psychologist by training, and his work address behavioral medicine and health outcomes (e.g., weight management). Although he has one publication that addresses sleep quality, it is not suicide-related.
- Consultant Leslie Ann Daline Brick, Ph.D., is Assistant Professor of Psychiatry and Human Behavior, Alpert Medical School, Brown University, and Associate Director of the Quantitative Science Program. She specializes in design and analysis of intensively sampled data including EMA of suicide outcomes following psychiatric hospitalizations among adults.
- Co-I Sheri L. Johnson, Ph.D., is Professor of Psychology at UC Berkeley. Her work includes assessments of impulsivity and cognition among suicidal adolescent inpatients. She is the originator of the emotion-related impulsivity construct, which figures heavily in the proposed project.

Weaknesses

- The relevance of Dr. Van Allen’s experience is less clear.
- No one from Covenant Medical Center—the sole inpatient recruitment site, is on the research team, even in a consultative capacity. Investment of Covenant personnel may be insufficient.

3. Innovation:
Strengths

- This is well written and innovative application with a well specified analytic plan.
- Within-person assessment of multiple RDoC domains (arousal/regulation, cognition) and how they may predict emotional and psychological precipitants of suicide (e.g., thwarted belongingness, low connection, hopelessness, defeat) through ideation-to-action mechanisms is novel and inventive.
- Assessment across units of analysis is a strength.
- Adding sleep to other RDoC constructs is a strength.

Weaknesses

- Main effects of many of these constructs and measures have been assessed previously. Although testing interactive effects could be innovative, such tests are not proposed and have limited power (see below).

4. Approach:
Strengths

- The within-person analyses are well planned and well justified. As the authors state, most research conducted to date has evaluated between-persons effects.
- Given recruitment for SA and no specific psychiatric diagnosis, the sample will be transdiagnostic.
- The intensive sampling will provide good temporal resolution.
- The very high-risk nature of the sample will yield considerable SI.
- Proposed tasks and methods are already in use by the research team and will therefore be readily implemented.

Weaknesses

- The aims, as specified on the aims page, are overly general. It would not have been possible to understand the aims without first reading the Project Abstract.
- The emotion-related impulsivity construct, around which the theoretical rationale is in-part constructed, poses problems for suicide research, especially from an RDoC perspective. It combines aspects of positive valence systems (impulsivity) with arousal/regulatory systems (emotional reactivity/lability), reducing them to a single dimension. This appears antithetical to RFA objectives of evaluating multiple RDoC constructs and their interactions with one another. Impulsivity and emotion regulation/dysregulation have different neural substrates and physiological correlates, and neither taken alone show strong associations with suicide. Associations are much stronger for those who experience both traits (i.e., 2 dimensions). Other theories of SI/SAs separate impulsivity from emotional lability/dysregulation. In the context of this RFA, these theories could be useful, but they are not considered or mentioned.
- Negative affective experiences (NAEs) comprise a primary independent variable. At multiple junctures, the authors state that 7 will be measured, from which a latent variable will be extracted. However, the specific 7 NAEs do not appear to be listed. Are they referring to low connection, pain, hopelessness, thwarted belongingness, burdensomeness, defeat, and entrapment? Such is unclear, and some of these are latent variables themselves. This is unclear from what's written.
- Prediction of suicide attempts across 28 days among 130 adults could be quite difficult. The authors do not present either (a) expected rates of SAs over this timespan or relatedly (b) effect sizes. In the power analysis, the Monte Carlo simulations address SI—not SAs—which occur less frequently.
- When SAs do occur, which will be followed by hospitalizations, how will participants' data be modelled? These participants will be censored out. Will they then be re-recruited?
- Also concerning power, the full model links (a) emotional suffering to (b) SI through (c) emotional suffering (Aim 1). Aims 2 and 3 link (a) sleep disturbance to (b) SA through (c) SI, moderated by (d) inhibitory control and (e) emotion-related impulsivity. Two comments: (1) in such moderated mediating analyses, variables can often be interchanged in the temporal sequence with reasonable fit either way, and (2) prediction of attempts relies on a significant higher-order interaction effect. Although the authors' power analysis does not include interactions, testing interactions among RDoC constructs is an RFA goal. Interaction effects are likely to be ordinal and of limited effect size given that all participants are hospitalized with histories of SI/SA. Sufficient power to detect complex ordinal interactions often requires several hundred to thousands of participants (there are many references in the literature). Thus, even if the authors' hypotheses are well-founded, they may fail to find predicted associations. Thus, the power analyses are highly optimistic.
- Minor note: The authors state that, "Psychological pain is robustly associated with suicide." This is a major overstatement. Psychological pain has very low sensitivity, low specificity, and low predictive power to suicide.
- As noted above, sex differences in self-reported precipitants of SA are well-known. Sex differences in other independent variables (e.g., impulsivity, affective lability, inhibitory control)

are also expected and often large. These are not discussed. Unaccounted for, sex differences these will add noise to an already underpowered design (see above).

- SUDs are not a rule-out and there is no plan to assess or track substance use and abuse—a major contributor to SA.
- Data provided on participant flow raise concerns about timely recruitment in such a short interval.

5. Environment:
Strengths

- The environment is deemed strong. The Texas Tech Psychology Department supports doctoral programs in clinical psychology and several other subdisciplines. Several faculty publish suicide-related research, and serval hold federal grants.
- The department supports Dr. Victor as an early career investigator; her faculty mentor (Dr. Jason Van Allen) is co-I.

Weaknesses

- Nothing about the Texas Tech University Health Sciences Center appears Under Facilities and Resources. There is a lack of information explaining the relationship between Texas Tech Psychiatry and Covenant's Behavioral Health Service. Existing collaborative relations between departments, if any, are unclear. Establishing new collaborations can be time consuming and effortful, making it difficult to pull-off this kind of work in only two years.
- Along similar lines, the following description in institutional support is concerning: “Dr. Victor has communicated directly with the Chief Medical Officer of Covenant Health, who is supportive of this research, as well as the Nurse Manager on the unit (Ms. Mary Giles), who has agreed to provide logistical and other support for the project. Dr. Victor and Ms. Giles have discussed the proposed project in depth and have begun the process of informing additional frontline staff about the project.” What is the PI’s relationship to Covenant?
- Although there is a letter of support from the Chief Medical Officer of Covenant Health, 68 unique patients per month may not be enough to accomplish the proposed research in two years. The authors reference a patient flow of 12.5 people per month who engage in SI/SA. If everything goes perfectly, it would take a year to recruit. How many of these patients will be ruled out for psychosis?

Protections for Human Subjects:
Acceptable Risks and/or Adequate Protections

- The authors provide an extensive plan for management of suicide risk and adverse events.

Data and Safety Monitoring Plan (Applicable for Clinical Trials Only):
Not Applicable (No Clinical Trials)

Inclusion Plans:

- Sex/Gender: Distribution justified scientifically.
- Race/Ethnicity: Distribution justified scientifically.
- Inclusion/Exclusion Based on Age: Distribution justified scientifically.
- Sex balance is 50/50. 46% of the sample will be Latinx.

Biohazards:
Not Applicable (No Biohazards)

Applications from Foreign Organizations:
Not Applicable (No Foreign Organizations)

Select Agents:
Not Applicable (No Select Agents)

Resource Sharing Plans:
Not Applicable (No Relevant Resources)

Authentication of Key Biological and/or Chemical Resources:
Not Applicable (No Relevant Resources)

Budget and Period of Support:
Recommend as Requested

- There is ambiguity regarding the consulting relationship between the PI and Dr. Brick. Dr. Brick's letter states $125/hour, whereas the budget states $5,000 flat fee/year, with little elaboration. This equates to 40 hours/year, which is reasonable given the importance of her role. Greater clarity would be helpful.

CRITIQUE 2

Significance: 4
Investigator(s): 3
Innovation: 5
Approach: 5
Environment: 1

Overall Impact:
This project addresses the goals of the FOA in that it addresses proximal risk through EMA, and impulsivity tasks and standard measures. The PI is accomplished and productive. The project's innovation is limited because the proposed factors have already been shown to be related to suicidal behavior. It is unclear how this will add to the literature. There are a number of methodological issues, the most significant one is that the follow-up suicide attempt rate, upon which two aims are based, will be very low, perhaps only 4-6, at most 10.

1. Significance:
Strengths

- The project addresses the goals of the FOA in that it addresses proximal risks to suicidal behavior.

Weaknesses

- It is unclear how the results of this study will move the field further. Sleep disturbance has been shown to be related to suicidal behavior; same for impulsivity and psychache.

2. Investigator(s):
Strengths

- The PI is young, very productive investigator who has conducted research on STBs and EMA and is well-suited to conducting this research.

Weaknesses

- EMA data analytic skills seem lacking. Dr. Brick who will be the data analyst on EMA has only one publication on EMA, none as first author.
- While Dr. Van Allen is accomplished research, his role and relevant research experience is unclear.
- Sleep expert consultation, particularly in relation to the strengths and weaknesses of using actigraphy to measure sleep disturbance, is missing.

3. Innovation:

Strengths
- Use of EMA in recently discharged suicidal patients.

Weaknesses
- Constructs being investigated have already been shown to be proximal risks to suicidal behavior; unclear how this project moves the field further.

4. Approach:
Strengths
- Well-designed procedures.

Weaknesses
- Unclear how psychache will be assessed through EMA; seems that only baseline measurement is done. How will the proposed mediation analyses be done?
- The methodological challenges and accuracy of the use of actigraphy for measuring sleep disturbance is not discussed.
- Inclusion criteria are not specific enough. SI or SB is listed as an inclusion criterion. What level of SI is required? Need to specify. What proportions for SI vs SB are required? As written, the study allows for 100% recruitment of individuals who exhibit SI without SB. This will affect the SA outcome rate. In order to address this questions this project asks, recent SB is needed or at least a significant proportion of participants.
- SI and SAs/SBs are primary outcomes. While the study will have sufficient SI in follow-up to have power to conduct the proposed analyses, the same cannot be said for SA. Aims 2 and 3 have SA as a primary outcome. In the power analysis, the PI states that, based on a recent study the rate of SA will be 10%. In fact, this is unlikely. The article cited found about a 10% rate of SBs, that includes interrupted and aborted attempts. According the article cited, 6.5% had an SA in follow-up. Furthermore, the length of follow-up in that study was 8 weeks for most of the patients (a minority were followed for four weeks). The current study has only a four week follow-up. Even in the best case scenario, with a 10% rate of SA, there would only be 10 SAs in follow-up. In the worst case scenario using the 6.5% rate and shorter follow-up, there would likely be somewhere between 4-6 SAs. Basing two of the major hypotheses on these numbers makes the study aims not testable.
- Sample size too small given above comment.

5. Environment:
Strengths
- Environment is strong; applicant provides support to demonstrate that recruitment is feasible.

Weaknesses
- None.

Protections for Human Subjects:
Acceptable Risks and/or Adequate Protections
Data and Safety Monitoring Plan (Applicable for Clinical Trials Only):
Not Applicable (No Clinical Trials)

Inclusion Plans:
- Sex/Gender: Distribution justified scientifically.
- Race/Ethnicity: Distribution justified scientifically.
- For NIH-Defined Phase III trials, Plans for valid design and analysis: Not applicable.
- Inclusion/Exclusion Based on Age: Distribution justified scientifically.

Biohazards:
Not Applicable (No Biohazards)

Applications from Foreign Organizations:
Not Applicable (No Foreign Organizations)

Select Agents:
Not Applicable (No Select Agents)

Resource Sharing Plans:
Acceptable

Authentication of Key Biological and/or Chemical Resources:
Not Applicable (No Relevant Resources)

Budget and Period of Support:
Recommend as Requested

CRITIQUE 3

Significance: 2
Investigator(s): 3
Innovation: 2
Approach: 3
Environment: 2

Overall Impact:
The proposed prospective assessment (time lagged approach) of proximal risk for suicide in terms of sleep disturbance, negative affect, inhibitory control and impulsivity is central to the understanding of the factors that contribute to STB. The model proffered (while a bit under articulated), the proposed experiment, and the team are good, if not exceptional. The application addresses the key goals of the RFA.

Concerns are that: 1) the sleep component of the model and experiment need work and the team needs a sleep expert; 2) the plan for recruitment (while a good approach) needs evidence that it can produce 100-130 enrolled subjects in 18 months (and a detailed backup plan is missing); and 3) a safety plan needs to be up front and center (presaged in abstract and specific aims, detailed in research plan and fully explicated in the human subjects). The model does not adequately take into account the ideas proffered by sleep researchers. Assurances and demonstrations are needed that the group has sufficient expertise to conduct actigraphy (both data acquisition and analysis). The proposed project needs to use as its' core retrospective sleep tools, not the PSQI and the GSAQ, but the ISI and the Sleep-50 or HSDQ or the SDS-CL-25. The PSQI can be a supplemental, but not a primary, measure.

1. Significance:
Strengths
- The proposed prospective assessment (time lagged approach) of proximal risk for suicide in terms of sleep disturbance, negative affect, inhibitory control and impulsivity is central to the understanding of the factors that contribute to STB. The application addresses the key goals of the RFA.

Weaknesses
- As noted above, the model does not adequately take into account the ideas proffered by sleep researchers.

2. Investigator(s):
Strengths
- The lead investigator (Dr. Victor) has excellent expertise in suicide research. She has assembled a strong team with expertise in the content area, grantsmanship, and experience re: study implementation.

Weaknesses
- While an R21 application and thus appropriate for less experienced investigator, Dr. Victor has no experience as an NIH PI.
- The application lacks an investigator who specializes in sleep and depression, or sleep and suicidality.

3. Innovation:
Strengths
- Given the RFA, the proposed project is not (per se) innovative. This said. there is absolutely a critical need to examine how day-to-day changes in negative affect, inhibitory control, and sleep (as a moderator of these vulnerabilities) give rise to STBs "at the within-person level". Taking into account the time lag between precipitant and consequent events is innovative as is the methodological innovation re: using an idiographic measure/definition of short sleep.

Weaknesses
- None Noted

4. Approach:
Strengths
- 130 Inpatient Adults studies following discharge from IPH. This is an ideal size sample and an ideal group in that the target cohort is an at risk group. More an at risk group studied at a time of high vulnerability (the month following discharge).
- The plan to recruit from inpatient units seems ideal, especially given the use of onsite recruiters and screening. Further, the investigators do a nice job of mentioning recruitment throughout the document, starting with the facilities page.
- The prospective assessment plan with EMA and actigraphy is a strength.

Weaknesses
- Given the region in which the study will be conducted, it would have been useful to highlight the access to Latino subjects and perhaps to propose targeted recruitment so that the study oversamples this minority to allow for exploratory analyses re: "race/ethnicity."
- Regarding recruitment, the census of available patients from covenant health (68/month for ~ 800 IP admissions per year) seems reasonable, but it's hard to know what proportion of this patients are appropriate (eligible) and what proportion of the willing could be enrolled. While hard numbers in this regard would be helpful, evaluating this in the context of an R21 is a reasonable goal. This said, the application lacks an adequate backup plan re: recruitment. The letter of support from Covenant health is appreciated, but it lacks specific information about patient flow, subject eligibility and the prospects for recruitment 130 individuals in 18 months.
- There is a lack of details regarding EMA. The prospective assessment of negative affect experiences, seems focused on affect without assessing for negative life events. Both could be assessed using EMA measures (in addition to full on instruments). This may have been the plan, but not enough detail was given re: the EMA measures. More, it was not clear if and how the other core concepts are to be measured prospectively with EMA.
- The application includes the use of out of date sleep measures. Emblematic of the absolute need for a sleep expert was the investigators' use of, and rationale for, the administration of the PSQI and HSAQ. Both instruments, while the first of their types, are no longer first line measures (not because of their age, but because the PSQI is a poor measure of anything in specific and especially sleep duration and/or sleep continuity disturbance. The HSAQ, while also one of the first of its type, only screens for 4 sleep disorders). While highly touted by some,

there are more comprehensive screeners: The Sleep-50; The HSDQ; or most recently the SDS-CL-25.

- The definition of short sleep duration: while the by subject approach to the definition of short sleep is a positive, it may be that TST is, for some subjects, low following hospitalization and/or for some subjects low “by nature.” It is likely best to categorize both trait and state sleep duration in addition to daily lows relative to 28 day means.
- The application does not do a good job of overtly and repeatedly acknowledging that recruiting at risk subjects requires a safety plan, for example brief descriptions and/or “flags” in the summary documents [abstract, aims, and specific aims], a larger explication in the research plan, and a “full on” description of the safety plan in Human Subjects section.
- Some claims are not accompanied by specifics and/or citations. For example, “Few NIMH-funded studies have examined contributors to short-term, within-person changes in suicidal ideation and odds of suicidal behavior.” In this instance, hard data could and should be provided (can be done using NIH Reporter). Some technical terms are not defined at first mention. For example, the term “psychache” is used early on without definition or citations.
- The abstract and specific aims sections required more information regarding the study design (e.g., info re: the number of subjects, the sample composition (racially diverse), primary DVs, etc.).

5. Environment:
Strengths

- No comment.

Weaknesses

- No comment.

Protections for Human Subjects:
Unacceptable Risks and/or Inadequate Protections

- The application does not do a good job of overtly and repeatedly acknowledging that recruiting at risk subjects requires a safety plan, for example brief descriptions and/or “flags” in the summary documents [abstract, aims, and specific aims], a larger explication in the research plan, and a “full on” description of the safety plan in Human Subjects section.

Data and Safety Monitoring Plan (Applicable for Clinical Trials Only):
Acceptable

Inclusion Plans:

- Sex/Gender: Distribution not justified scientifically.
- Race/Ethnicity: Distribution not justified scientifically.
- Inclusion/Exclusion Based on Age: Distribution not justified scientifically.
- There is a lack of information provided in the research strategy about sample composition with respect to sex and age. The investigators do highlight that their sample will likely be comprised of a large percent of Latinx individuals. This is a major strength of the application, one that should have been featured.

Biohazards:
Not Applicable (No Biohazards)

Applications from Foreign Organizations:
Not Applicable (No Foreign Organizations)

Select Agents:
Not Applicable (No Select Agents)

Resource Sharing Plans:
Not Applicable (No Relevant Resources)

- Not sure that this applied to R21s. If it does, there does not appear to be a data sharing section.

Authentication of Key Biological and/or Chemical Resources:
Not Applicable (No Relevant Resources)

Budget and Period of Support:
Recommend as Requested

THE FOLLOWING SECTIONS WERE PREPARED BY THE SCIENTIFIC REVIEW OFFICER TO SUMMARIZE THE OUTCOME OF DISCUSSIONS OF THE REVIEW COMMITTEE, OR REVIEWERS' WRITTEN CRITIQUES, ON THE FOLLOWING ISSUES:

PROTECTION OF HUMAN SUBJECTS: ACCEPTABLE

INCLUSION OF WOMEN PLAN: ACCEPTABLE

INCLUSION OF MINORITIES PLAN: ACCEPTABLE

INCLUSION ACROSS THE LIFESPAN: ACCEPTABLE

SCIENTIFIC REVIEW OFFICER'S NOTES: Sex as a biological variable is not adequately considered. The authors do not adequately consider sex differences in self-reported precipitants of suicide ideation and suicide attempt.

COMMITTEE BUDGET RECOMMENDATIONS: The budget was recommended as requested. As a comment, there is ambiguity regarding the consulting relationship between the PI and Dr. Brick. Dr. Brick's letter states $125/hour, whereas the budget states $5,000 flat fee/year, with little elaboration. This equates to 40 hours/year, which is reasonable given the importance of her role. Greater clarity would be helpful.

Footnotes for 1 R21 MH124794-01; PI Name: Victor, Sarah Elizabeth

NIH has modified its policy regarding the receipt of resubmissions (amended applications).See Guide Notice NOT-OD-18-197 at https://grants.nih.gov/grants/guide/notice-files/NOT-OD-18-197.html. The impact/priority score is calculated after discussion of an application by averaging the overall scores (1-9) given by all voting reviewers on the committee and multiplying by 10. The criterion scores are submitted prior to the meeting by the individual reviewers assigned to an application, and are not discussed specifically at the review meeting or calculated into the overall impact score. Some applications also receive a percentile ranking. For details on the review process, see http://grants.nih.gov/grants/peer_review_process.htm#scoring.

MEETING ROSTER

National Institute of Mental Health Special Emphasis Panel
NATIONAL INSTITUTE OF MENTAL HEALTH
Dysregulation and Proximal Risk for Suicide Review Meeting (R01 & R21)

ZMH1 ERB-S (07)
07/09/2020

Notice of NIH Policy to All Applicants: Meeting rosters are provided for information purposes only. Applicant investigators and institutional officials must not communicate directly with study section members about an application before or after the review. Failure to observe this policy will create a serious breach of integrity in the peer review process, and may lead to actions outlined in NOT-OD-14-073 at https://grants.nih.gov/grants/guide/notice-files/NOT-OD-14-073.html and NOT-OD-15-106 at https://grants.nih.gov/grants/guide/notice-files/NOT-OD-15-106.html, including removal of the application from immediate review.

CHAIRPERSON(S)

AREAN, PATRICIA A., PHD
PROFESSOR
DEPARTMENT OF PSYCHIATRY AND BEHAVIORAL SCIENCES
SCHOOL OF MEDICINE
UNIVERSITY OF WASHINGTON
SEATTLE, WA 98102

MEMBERS

ANSELL, EMILY B, PHD
ASSOCIATE PROFESSOR
DEPARTMENT OF BIOBEHAVIORAL HEALTH
THE PENNSYLVANIA STATE UNIVERSITY
UNIVERSITY PARK, PA 16802

BEAUCHAINE, THEODORE PATRICK, PHD
PROFESSOR
DEPARTMENT OF PSYCHOLOGY
THE OHIO STATE UNIVERSITY
COLUMBUS, OH 43210

DEPP, COLIN A., PHD
PROFESSOR
DEPARTMENT OF PSYCHIATRY
SCHOOL OF MEDICINE
UNIVERSITY OF CALIFORNIA, SAN DIEGO
LA JOLLA, CA 92093

DICHTER, GABRIEL S, PHD
PROFESSOR OF PSYCHIATRY AND PSYCHOLOGY & NEUROSCIENCE
ASSOCIATE DIRECTOR, UNC INTELLECTUAL AND DEVELOPMENTAL DISABILITIES RESEARCH CENTER
SCHOOL OF MEDICINE
UNIVERSITY OF NORTH CAROLINA AT CHAPEL HILL
CHAPEL HILL, NC 27599

GUO, YING, PHD
PROFESSOR
DEPARTMENT OF BIOSTATISTICS AND BIOINFORMATICS
ROLLINS SCHOOL OF PUBLIC HEALTH
EMORY UNIVERSITY
ATLANTA, GA 30322

HAJCAK, GREG, PHD
PROFESSOR
DEPARTMENT OF PSYCHOLOGY
COLLEGE OF ARTS AND SCIENCES
FLORIDA STATE UNIVERSITY
TALLAHASSEE, FL 32306

HAZLETT, ERIN A., PHD
PROFESSOR
DEPARTMENTS OF PSYCHIATRY AND NEUROSCIENCE
ICAHN SCHOOL OF MEDICINE AT MOUNT SINAI
NEW YORK, NY 10029

KENSINGER, ELIZABETH ANN, PHD
PROFESSOR
DEPARTMENT OF PSYCHOLOGY
BOSTON COLLEGE
CHESTNUT HILL, MA 02467

KLIMES-DOUGAN, BONNIE, PHD
ASSOCIATE PROFESSOR
DEPT OF PSYCHOLOGY
UNIVERSITY OF MINNESOTA
MINNEAPOLIS, MN 55455

KRYSTAL, ANDREW D, MD
VICE CHAIR FOR RESEARCH
DEPARTMENT OF PSYCHIATRY
SCHOOL OF MEDICINE
UNIVERSITY OF CALIFORNIA, SAN FRANCISCO
SAN FRANCISCO, CA 94143

LEUCHTER, ANDREW F, MD
PROFESSOR
DEPARTMENT OF PSYCHIATRY AND
BIOBEHAVIORAL SCIENCES
DAVID GEFFEN SCHOOL OF MEDICINE
UNIVERSITY OF CALIFORNIA, LOS ANGELES
LOS ANGELES, CA 90095

LOCK, JAMES D, MD, PHD
PROFESSOR
DEPARTMENT OF PSYCHIATRY AND
BEHAVIORAL MEDICINE
LUCILE SALTER PACKARD CHILDREN'S HOSPITAL
STANFORD UNIVERSITY SCHOOL OF MEDICINE
PALO ALTO, CA 94304

MELHEM, NADINE M., PHD
ASSOCIATE PROFESSOR
DEPARTMENT OF PSYCHIATRY
UNIVERISTY OF PITTSBURGH
PITTSBURGH, PA 15213

MIRANDA, REGINA, PHD
PROFESSOR
DEPARTMENT OF PSYCHOLOGY
HUNTER COLLEGE
NEW YORK, NY 10065

NOCK, MATTHEW K, PHD
PROFESSOR
HARVARD UNIVERSITY
DEPARTMENT OF PSYCHOLOGY
CAMBRIDGE, MA 01238

ONNELA, JUKKA-PEKKA, DSC
ASSOCIATE PROFESSOR
DEPARTMENT OF BIOSTATISTICS
HARVARD SCHOOL OF PUBLIC HEALTH
BOSTON, MA 02115

PERLIS, MICHAEL LLOYD, PHD
ASSOCIATE PROFESSOR
DEPARTMENT OF PSYCHIATRY
UNIVERSITY OF PENNSYLVANIA
PHILADELPHIA, PA 19104

STANLEY, BARBARA, PHD
PROFESSOR
DEPARTMENT OF PSYCHIATRY
COLUMBIA UNIVERSITY
NEW YORK, NY 10032

STEVENS, MICHAEL C, PHD
DIRECTOR, CHILD AND ADOLESCENT RESEARCH
OLIN NEUROPSYCHIATRY RESEARCH CENTER
HARTFORD HOSPITAL INSTITUTE OF LIVING
HARTFORD, CT 06106

WILDES, JENNIFER E., PHD
ASSOCIATE PROFESSOR
DEPARTMENT OF PSYCHIATRY
UNIVERSITY OF CHICAGO SCHOOL OF MEDICINE
CHICAGO, IL 60637

SCIENTIFIC REVIEW OFFICER

GARCIA, REBECCA STEINER, PHD
SCIENTIFIC REVIEW OFFICER
DIVISION OF EXTRAMURAL ACTIVITIES
NATIONAL INSTITUTE OF MENTAL HEALTH
NATIONAL INSTITUTES OF HEALTH
BETHESDA, MD 20892

EXTRAMURAL SUPPORT ASSISTANT

THAI, JULIE PHAM
EXTRAMURAL SUPPORT ASSISTANT (CONTRACTOR)
NATIONAL INSTITUTE OF MENTAL HEALTH
NEUROSCIENCE CENTER
BETHESDA, MD 20892

Consultants are required to absent themselves from the room during the review of any application if their presence would constitute or appear to constitute a conflict of interest.
